# An Iron-Mimicking, Trojan Horse-Entering Fungi—Has the Time Come for Molecular Imaging of Fungal Infections?

**DOI:** 10.1371/journal.ppat.1004568

**Published:** 2015-01-29

**Authors:** Hubertus Haas, Milos Petrik, Clemens Decristoforo

**Affiliations:** 1 Division of Molecular Biology/Biocenter, Innsbruck Medical University, Innsbruck, Austria; 2 Institute of Molecular and Translational Medicine, Faculty of Medicine and Dentistry, Palacky University, Olomouc, Czech Republic; 3 Clinical Department of Nuclear Medicine, Innsbruck Medical University, Innsbruck, Austria; Duke University Medical Center, UNITED STATES

## Introduction

Despite recent advancements in the diagnosis and management of fungal infections [1], invasive fungal diseases remain a major cause of morbidity and mortality in immunocompromised patients and are major drivers of elevated healthcare costs [2]. In this context, early diagnosis is a key factor. However, current diagnostic approaches, including laboratory tests and computer tomography, have limitations, especially in terms of sensitivity and specificity [3]. Therefore, empirical therapy has often evolved as the standard of care, irrespective of the immediate and long-term consequences in terms of cost, development of drug resistance, or toxicity [4].

An exceptional challenge is the development of imaging modalities providing not only high specificity and sensitivity but also localization of the infection site. In particular, nuclear medicine imaging techniques using radiolabelled probes (radiotracers) have the potential to specifically target the underlying pathophysiological mechanisms of the pathogen leading to molecular localization of the infection site in patients.

Traditionally applied in the context of planar scintigraphy and single photon emission tomography (SPECT) in the past decade, Positron-Emission Tomography (PET) has evolved as a major clinical imaging technique, particularly in oncology [5]. This technology provides improved sensitivity and resolution based on the coincidence detection of photons emitted from radionuclei resulting from annihilation of positrons. Its tremendous success in oncology is mainly based on 2-[^18^F] fluorodeoxyglucose specifically accumulating in cells in dependence of their glucose consumption [6]. In this process, termed “molecular trapping,” the radiolabelled glucose molecule is actively transported into the cell, followed by its phosphorylation. The incorporated Fluor blocks further metabolic processing and traps the radionuclide ^18^F inside the cell, leading to an intense radioactive signal in affected cells. Other clinically used PET probes, such as [^18^F]-3′-fluoro-3′-deoxy-L-thymidine, [^18^F]-choline derivatives, or radiolabelled peptides, such as ^68^Ga-DOTATOC, show a similar trapping mechanism based on initial active transport in, or receptor-specific recognition by, diseased cells with promising clinical applications in oncology [7]. Besides accumulation in the target, favorable pharmacokinetics of radiotracers, such as rapid transport to and low retention in non-target sites/cells as well as efficient elimination from the body, ideally via renal excretion, are required. These features allow early imaging with short-lived radionuclides such as ^18^F with a half-life of 110 minutes, resulting in a low radiation burden for the patient.

Therefore, a radiotracer for specific imaging of fungal infections should ideally fulfill similar criteria: specific accumulation in the pathogen combined with favorable pharmacokinetics, including rapid elimination from healthy tissue. Ideally, the pathogen should recognize the radiotracer as an apparent molecule of interest, boosting its active uptake and accumulation.

## Attempts to Image Fungal Infections with Radiolabeled Probes

A number of non-specific radiotracers have been applied for imaging of infections. These include ^111^In- or ^99m^Tc-labelled leucocytes, ^99m^Tc-anti-granulocyte antibody, ^99m^Tc-diphosphonates in the context of bone scanning, ^67^Ga-citrate, and even 2-[^18^F]-fluorodeoxyglucose [8]. These probes target predominantly secondary effects of infection, such as increased blood flow and vascular permeability, activated endothelial cells, or polymorphonuclear cell migration. Therefore, several attempts have been made to develop more specific radiotracers for fungal infections [9]. A widely studied group of compounds are antimicrobial peptides, which typically interact through their cationic domains with the anionic surface of microorganisms, leading to antimicrobial activity. Particularly, ^99m^Tc-labelled ubiquicidin and human lactoferrin derivatives for SPECT have been developed with promising results in patients with bacterial infections, but no specificity for fungal infections preclinically. Another approach was the use of ^99m^Tc-labelled fluconazole, a widely used triazole antifungal drug that inhibits ergosterol biosynthesis, which, however, accumulated poorly in *A. fumigatus* in a murine infections model [9]. ^123^I-labelling of chitinase and ^99m^Tc-labelling of the chitin-binding protein CBP21 have been described as specific imaging agents for fungal infections, but were never translated to human studies [9]. Moreover, ^99m^Tc-labelled polyethylene glycol (PEG)-liposomes and ^99m^Tc-interleukin 8 showed interesting preclinical results, but these compounds show unfavorable pharmacokinetics [9]. All these agents lack specificity for fungal pathogens and, therefore, have not found their way into clinical fungal infection imaging.

## Attempts to Specifically Image Aspergillosis

Among fungal pathogens, *Aspergillus fumigatus* is still the most common opportunistic mold pathogen infecting humans with a fourfold increase of invasive pulmonary aspergillosis over the last 30 years [10]. Recently, interesting approaches to develop a radiotracer specific for *A. fumigatus* have been reported. ^99m^Tc-labeled phosphorodiamidate morpholino (MORF) oligomers that hybridize to the fungal ribosomal RNA were found to accumulate in infected lungs as compared to a control construct [11]. The cyclic peptide c(CGGRLGPFC)-NH2, identified by phage display technology to bind in vitro to the surface of conidia and hyphae of *A. fumigatus*, is able to image *A. fumigatus* lung infection in a mouse model using SPECT, when labeled with ^111^In [12]. Moreover, an *Aspergillus*-specific monoclonal antibody used for serodiagnosis of aspergillosis is currently being examined for its potential in PET imaging [13]. These promising initial results indicate the potential to specifically target *A. fumigatus*. However, all of these methods appear to have one major drawback: namely, a lack of active uptake by the pathogen leading to signal intensification at the infection site. This has led us to investigate the potential of the siderophore system as a target for a radiotracer with high specificity and sensitivity for fungal infections.

## Siderophore Metabolism of *A. Fumigatus* as the Basis for Molecular Imaging

**Figure 1:**
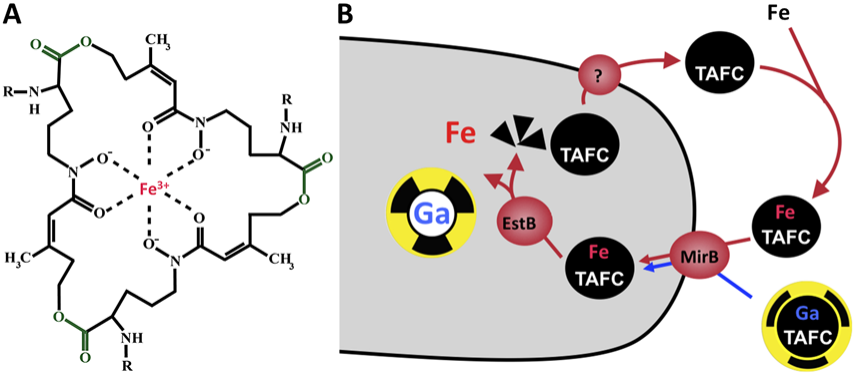
Siderophore mediated-iron uptake in *A. fumigatus*. (A) FSC/TAFC is shown in the ferri-form; the ester bonds separating the three *N* ^5^-acetyl-/*N* ^5^-anhydromevalonyl-*N* ^5^-hydroxyornithine residues are shown in green; for TAFC-based nuclear imaging, the iron (shown in red) is replaced by ^68^Ga. FSC, R = H; TAFC, R = acetyl. (B) TAFC-mediated uptake of iron and gallium into fungal hypha.

During infection, pathogens encounter an essentially iron-free environment as the available iron is tightly sequestered by host proteins, e.g., hemoglobin, transferrin, lactoferrin, and ferritin [14]. Consequently, pathogens had to evolve mechanisms for “stealing” host iron. *A. fumigatus* possesses two high-affinity iron uptake systems, reductive iron assimilation and siderophore-mediated iron acquisition [15]. Both systems are transcriptionally upregulated during iron starvation in vitro as well as in vivo in a murine model for pulmonary aspergillosis confirming that *A. fumigatus* faces iron starvation conditions during infection [16, 17]. In the murine infection model, genetic inactivation of siderophore biosynthesis, but not of reductive iron assimilation, attenuated virulence of *A. fumigatus*, demonstrating that siderophore-mediated iron assimilation plays the major role for virulence [15]. Siderophores are ferric iron-specific chelators secreted by a diverse range of bacteria and fungi, displaying a variety of structures depending on the producer [18]. With iron binding constants of 10^20^–10^50^, siderophores are able to sequester iron from iron-binding host proteins such as transferrin [19]. *A. fumigatus* secretes two hydroxamate-type siderophores (Fig. 1A), fusarinine C (FSC), which consists of three *N* ^5^-anhydromevalonyl-*N* ^5^-hydroxyornithine residues cyclically linked by ester bonds, and its *N^2^*-acetylated derivative triacetylfusarinine C (TAFC). FSC/TAFC synthesis involves six enzymes dedicated solely to siderophore biosynthesis [20–22]. The cellular export mechanism for siderophores remains to be characterized. After chelation of iron, the uptake of ferri-siderophores is mediated by specific transporters, which belong to the Siderophore Iron Transporter (SIT) subfamily of the major facilitator protein superfamily [23]. *A. fumigatus* possesses seven SIT proteins, five of which are transcriptionally upregulated by iron starvation. Among these, MirB was identified as the transporter for TAFC while the FSC transporter remains to be identified [24, 25]. In the cell, the trilactone rings of TAFC and FSC are hydrolyzed by specific esterases [26, 27]. The released iron is transferred to the metabolism or stored either in the intracellular siderophore ferricrocin or within the vacuole [28]. A scheme of TAFC-mediated iron uptake is shown in Fig. 1B.

Most fungal species produce siderophores, but there are notable exceptions, such as the fungal model species *Saccharomyces cerevisiae*, *Cryptococcus neoformans*, and *Candida* spp. [23]. SIT members are encoded by all fungal species. Consistently, non-siderophore-producing species were also shown to use such transporters for uptake of xenosiderophores, siderophores produced by other microorganisms [29]. Moreover, siderophore-producing fungal species are also able to take up xenosiderophores [30]. The siderophore system is confined to the fungal and bacterial kingdoms, i.e., the enzymes and transporters involved in biosynthesis and uptake of siderophores are not present in vertebrate cells.

## A Trojan Horse for the Iron-Searching Pathogen

Siderophore transporters represent a promising target for molecular imaging approaches in fungal infections due to the following reasons: (i) they are highly upregulated during infection, which is underlined by their crucial role in virulence of *A. fumigatus*; (ii) these transporters are not present in human cells and therefore their specific substrates do not interact with the human physiology; (iii) the energy-dependent active uptake leads to accumulation of a (radio)labeled substrate in the pathogen; (iv) the low molecular mass of siderophores (e.g., the *M_r_*of desferri-TAFC is 853 Da) has the advantage of rapid diffusion from the circulation into infected tissues; (v) the chemical features of TAFC, but not all siderophores, result in low binding to serum components, which is crucial for a high signal-to-noise ratio and rapid clearance from non-target tissue and elimination from the body via renal excretion; (vi) radiolabelling of siderophores can be achieved easily by replacing Fe(III) in the siderophore with an iron-mimicking radionuclide. There is no iron isotope with suitable properties for imaging in terms of half-life and photon emission. However, it has been known for a long time that Ga(III) is an isosteric diamagnetic substitute for Fe(III), which has been used to characterize siderophore complexes and siderophore uptake, revealing indistinguishable uptake compared to the Fe(III) counterpart in *Ustilago sphaerogena* [31, 32]. Recently, the interest in ^68^Ga has increased tremendously with the establishment of PET as a clinical imaging modality [33]. ^68^Ga with a physical half-life of 68 minutes can be obtained from a ^68^Ge/^68^Ga generator without the requirement of cyclotron installations and exhibits a very low radiation burden to the patient, comparable or even lower to that of ^18^F, the most widely used radionuclide for PET.

**Figure 2:**
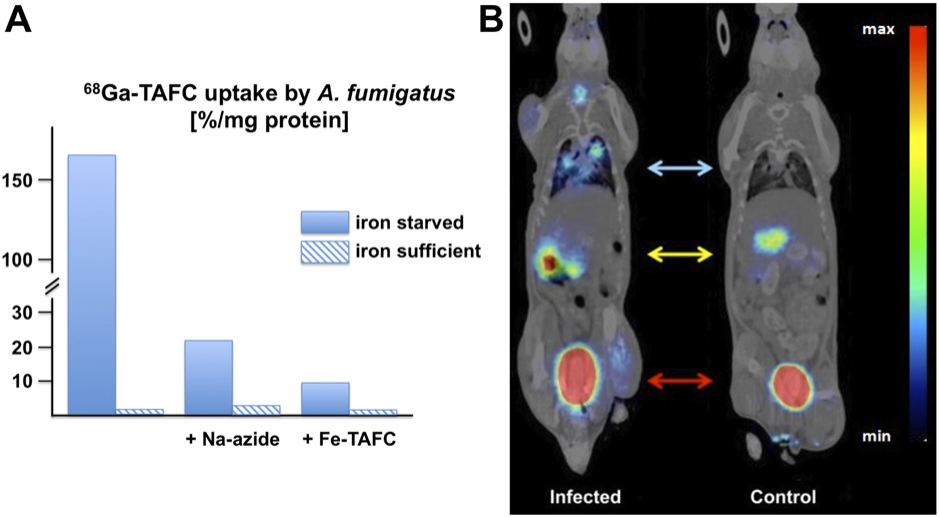
In vitro and in vivo uptake of ^68^Ga-TAFC by *A. fumigatus*. (A) *In-vitro* uptake of ^68^Ga-TAFC in *A. fumigatus* cultures, showing induction of uptake during iron starvation, energy-dependence, and saturation by excess of ferric TAFC. (B) Micro-PET/CT (Albira PET/SPECT/CT small animal imaging system, Bruker Biospin Corporation, Woodbridge, CT, USA) imaging of *A. fumigatus* (coronal slices) in a rat infection model 45 minutes post intravenous injection of ^68^Ga-TAFC showing clear accumulation (blue arrow) in infected lung tissue (M. Petrik, unpublished). Accumulation of ^68^Ga-TAFC in kidney (yellow arrow) and bladder (red arrow) is caused by rapid renal excretion of the tracer. The colors reflect the signal intensity increasing from blue to green, yellow and red.

In a proof of concept study [34], we demonstrated that desferri-siderophores, particularly TAFC, can be easily radiolabelled with ^68^Ga exhibiting favorable hydrophilic properties and high chemical stability. Uptake of ^68^Ga-TAFC in *A. fumigatus* was upregulated under iron starvation conditions and could be blocked with an excess of siderophore or NaN_3_, indicating specific and energy-dependent uptake (Fig. 2A). In vivo, in an aspergillosis rat model, ^68^Ga-TAFC was rapidly excreted, exclusively renally as intact complex, indicating excellent metabolic stability. Compared to healthy lungs, ^68^Ga-TAFC was taken up in a more than 20-fold higher concentration in infected lung tissue. A variety of different siderophores, including FSC, TAFC, coprogen, as well as various ferrichrome-type and ferroxamine-type-siderophores, displayed excellent ^68^Ga-radiolabeling [30]. However, only ^68^Ga-TAFC and ^68^Ga-ferrioxamine E (FOXE), a siderophore produced by various *Streptomycetes* species [35], displayed a good combination of fungal uptake in culture combined with high chemical and metabolic stability as well as suitable pharmacokinetics for imaging, i.e., rapid clearance from organs and circulation with predominant renal excretion. High contrast imaging of *A. fumigatus* pulmonary infection in a rat model could be achieved using Micro-PET/computed tomography (CT) technology, exhibiting pronounced accumulation of ^68^Ga-TAFC in infected areas (Fig. 2B). This was achieved already early after the onset of infection and increased with its severity and correlated with abnormal CT images [36]. Most vertebrates produce siderophore-scavenging proteins, termed siderocalins, to limit the growth of pathogens. The major siderocalin found in the murine and human blood, NGAL/Lcn2, does not recognize either TAFC nor FOXE [37] and, consequently, does not impede these tracers.

## Is the Trojan Horse Only Finding the Right Target?

Before using such a potentially powerful imaging weapon, potential limitations have to be addressed, such as the sensitivity and specificity of such a method. In a clinical setting, the iron status or antifungal medication of the patient may influence the activity of the siderophore system and the rate of fungal iron acquisition, thereby influencing the radiotracer uptake und consequently the sensitivity of this method. Patients who acquire fungal infections often suffer from iron overload (e.g., from blood transfusions), which is a major risk factor in the onset of the infection. In a preliminary study, no significant influence of iron overload of *A. fumigatus* infected rats on lung uptake was observed. The influence of pre- or concomitant medication still has to be addressed in a preclinical model.

Regarding specificity, the discrimination between sterile inflammation, tumors, and real infection is crucial. In this respect, significant accumulation of ^68^Ga TAFC was neither found in a sterile inflammation model nor in tumor cells [38]. As TAFC is produced by different fungal species, e.g., *A. nidulans* and *A. fumigatus*, and might be used as a xenosiderophore by non-produces, the specificity among microbial pathogens is another important issue. ^68^Ga-TAFC uptake studies revealed in vitro high uptake by *A. fumigatus*, considerable uptake by *Rhizopus oryzae* and *Fusarium solani*, but no significant uptake by *Aspergillus terreus, Aspergillus flavus*, *Candida albicans*, as well as the bacterial species *Pseudomonas aeruginosa*, *Klebsiella pneumoniae*, *Staphylococcus aureus*, or *Mycobacterium smegmatis*. In comparison, FOXE displayed the highest uptake by *A. fumigatus*, and considerable uptake by *A. terreus*, *A. flavus*, *Rhizopus oryzae*, *Fusarium solani*, as well as the bacterial species *Staphylococcus aureus*. In vivo, however, neither TAFC nor FOXE were found to accumulate in a rat *S. aureus* abscess model [38]. Taken together, TAFC and FOXE have overlapping species specificity with the best uptake observed for *A. fumigatus*, among all tested species. Nevertheless, further in vivo studies are required to clarify the species specificity. Moreover, the sensitivity of this approach compared to current diagnostic methods has to be addressed, ideally in a clinical setting.

## Conclusion

From these studies, we conclude that ^68^Ga-labeled siderophores, particularly ^68^Ga-TAFC, have a high potential to be used as fungal radiotracers: easily generated by direct radiolabeling of the desferri-form by incubation with a ^68^Ga-solution in a pharmaceutically compatible acetate buffer, ^68^Ga-TAFC displays high stability both in solution and in vivo, and rapidly reaches the infection site where the pathogen misinterprets the radiolabeled tracer as an iron source and accumulates this “Trojan horse.” The next major step forward in this attempt to provide a tool for molecular imaging of fungal infections will be the proof of concept in a clinical study that is currently in its planning phase. It will show whether targeting of a molecular process related to the pathophysiology of infection indeed will be successful to provide the clinical information required to establish Odysseus’ concept of the Trojan horse at the molecular level.
